# Baseline Association Between Remotely‐measured Cognitive Function and Blood Pressure in a Large, Multicenter Pragmatic Hybrid Effectiveness‐Implementation Trial

**DOI:** 10.1002/alz70858_104060

**Published:** 2025-12-26

**Authors:** Adam Parks, Xing Song, Kate Young, Jonathan D Mahnken, Sravani Chandaka, Diego R Mazzotti, Molly Conroy, Mark A. Supiano, Jeffrey M. Burns, Aditi Gupta

**Affiliations:** ^1^ University of Kansas Medical Center, Kansas City, KS, USA; ^2^ University of Missouri School of Medicine, Columbia, MO, USA; ^3^ University of Utah, Salt Lake City, UT, USA

## Abstract

**Background:**

Hypertension is a modifiable risk factor for dementia. Lowering blood pressure (BP) can slow cognitive decline and reduce the risk of dementia, including Alzheimer's disease (AD). Data on the association between BP and cognitive function in real‐world primary care clinics are sparse. Additionally, data on remote assessment of cognitive function are also limited.

**Method:**

Baseline data were analyzed from a larger multicenter pragmatic effectiveness‐implementation hybrid trial assessing the effect of a virtual hypertension program on BP control and cognitive decline. Using EHR, patients with high BP were automatically identified and randomized to usual care or the irtual collaborative care clinic (vCCC), a hypertension program which integrates self‐measured BP (SMBP), remote monitoring and management, and frequent patient‐provider communication. Cognitive function was measured using Telephone Cognitive Assessment (Tcog). Tcog is a standardized neuropsychological test battery that assesses cognitive function remotely via telephone and measures memory, executive functions, attention, and language. Eligible patients completed a baseline visit consisting of SMBP and Tcog. To explore the association between baseline BP and cognitive function, multiple regression analyses, with age and sex as covariates, were used.

**Result:**

Data from 1,000 older adults, mean (SD) age = 73.3 (5.8), 61% female, 88% White, 10% Black, who completed the baseline visit was analyzed. The mean (SD) systolic BP was 135 (17) mmHg. The mean (SD) global cognition score (MoCA‐22) was 18.4 (2.5). There was no association between baseline systolic BP and MoCA‐22 performance. Higher systolic BP was significantly associated with poorer performance in semantic fluency (β = ‐0.041, 95% CI: ‐0.069 to ‐0.013, *p* = 0.004) and verbal learning (β = ‐0.038, 95% CI: ‐0.075 to ‐0.0019, *p* = 0.039).

**Conclusion:**

Higher systolic BP is associated with poorer performance in semantic fluency and verbal learning, independent of age and sex, yet it is not associated with global cognitive function. These findings suggest that hypertension may selectively impact specific cognitive domains, particularly those related to language and memory. Furthermore, these abilities are often affected early in AD. Future analyses will explore whether lowering BP can slow cognitive decline in the broad patient population seen in the primary care setting.

